# Alzheimer’s Disease as a Membrane Disorder: Spatial Cross-Talk Among Beta-Amyloid Peptides, Nicotinic Acetylcholine Receptors and Lipid Rafts

**DOI:** 10.3389/fncel.2019.00309

**Published:** 2019-07-18

**Authors:** Camila Fabiani, Silvia S. Antollini

**Affiliations:** ^1^Instituto de Investigaciones Bioquímicas de Bahía Blanca CONICET-UNS, Bahía Blanca, Argentina; ^2^Departamento de Biología, Bioquímica y Farmacia, Universidad Nacional del Sur, Bahía Blanca, Argentina

**Keywords:** Alzheimer’s disease, Aβ peptide, nicotinic acetylcholine receptor, acetylcholinesterase, cell membranes, lipid rafts, cholesterol

## Abstract

Biological membranes show lateral and transverse asymmetric lipid distribution. Cholesterol (Chol) localizes in both hemilayers, but in the external one it is mostly condensed in lipid-ordered microdomains (raft domains), together with saturated phosphatidyl lipids and sphingolipids (including sphingomyelin and glycosphingolipids). Membrane asymmetries induce special membrane biophysical properties and behave as signals for several physiological and/or pathological processes. Alzheimer’s disease (AD) is associated with a perturbation in different membrane properties. Amyloid-β (Aβ) plaques and neurofibrillary tangles of tau protein together with neuroinflammation and neurodegeneration are the most characteristic cellular changes observed in this disease. The extracellular presence of Aβ peptides forming senile plaques, together with soluble oligomeric species of Aβ, are considered the major cause of the synaptic dysfunction of AD. The association between Aβ peptide and membrane lipids has been extensively studied. It has been postulated that Chol content and Chol distribution condition Aβ production and posterior accumulation in membranes and, hence, cell dysfunction. Several lines of evidence suggest that Aβ partitions in the cell membrane accumulate mostly in raft domains, the site where the cleavage of the precursor AβPP by β- and γ- secretase is also thought to occur. The main consequence of the pathogenesis of AD is the disruption of the cholinergic pathways in the cerebral cortex and in the basal forebrain. In parallel, the nicotinic acetylcholine receptor has been extensively linked to membrane properties. Since its transmembrane domain exhibits extensive contacts with the surrounding lipids, the acetylcholine receptor function is conditioned by its lipid microenvironment. The nicotinic acetylcholine receptor is present in high-density clusters in the cell membrane where it localizes mainly in lipid-ordered domains. Perturbations of sphingomyelin or cholesterol composition alter acetylcholine receptor location. Therefore, Aβ processing, Aβ partitioning, and acetylcholine receptor location and function can be manipulated by changes in membrane lipid biophysics. Understanding these mechanisms should provide insights into new therapeutic strategies for prevention and/or treatment of AD. Here, we discuss the implications of lipid-protein interactions at the cell membrane level in AD.

## Introduction

Biological membranes were, are, and will be complex, dynamic and controversial. Several different theories/models were postulated until the fluid-mosaic model was proposed by Singer and Nicolson (1972). This description of a biological membrane was very well accepted and gave light about membrane structure and membrane function. Although a lot of new information appeared in the following 40 years, the model was able to survive by absorbing some modifications, as it was emphasized by Nicolson (2014). Table 1 details and compares the most important features of the original fluid-mosaic model membrane (Singer and Nicolson, 1972) and the current vision of a membrane (Engelman, 2005; Bagatolli, 2010; Goñi, 2014; Nicolson, 2014).

**TABLE 1:** Comparison of the main membrane characteristics proposed by the Singer and Nicolson (1972) model and the current cell membrane vision (based on Engelman (2005), Bagatolli (2010), Goñi (2014), Nicolson (2014), and references there in).

**Singer and Nicolson (1972)**	**Today (2019)**
The membrane consists of a double layer of lipids (*bilayer*) in a *lamellar liquid-crystalline phase.* (A preliminary deviation of this concept was included in the original model: *“It is therefore not excluded that some significant fraction of the phospholipid (perhaps as much as 30 percent) is physically in a different state from the rest of the lipid.”*)	In certain membranes, other phases like *liquid-ordered phases* or *non-lamellar phases* as rhombohedral, tetragonal, inverted hexagonal and cubic phases fulfill physiologically important functions. These phenomena involve membrane phase changes that are possible because of the intrinsic deformability of the membrane. Examples of transient *non-lamellar phases* can be seen during membrane fusion where from two independent bilayers originates only one which involves the coalesce of two bilayers (Chernomordik and Zimmerberg, 1995; Tenchov et al., 2006) or during pore formation by proteins as Bax/colicin family proteins and actinoporins which involves the formation of non-lamellar (semi-toroidal or toroidal) lipidic structures (Gilbert et al., 2014; Gilbert, 2016).

The membrane is considered *flat.*	Membranes are usually *curved*, dynamically modulated by the geometry of both lipids and proteins, and require *asymmetry* between both hemilayers in order to support this membrane curvature (Mouritsen and Bloom, 1984; Epand et al., 1996; Zimmerberg and Gawrisch, 2006; Bagatolli, 2010).

The protein:lipid ratio is 1.5–4, and thus proteins play an important role in the membrane structure. However, lipids and proteins do not interact strongly. They are almost independent entities, without significant perturbation of the bilayer. (A preliminary deviation of this concept was proposed in the original model: *“if it is proposed that, while the largest portion of the phospholipid is in bilayer form and not strongly coupled to proteins in the membrane a small fraction of the lipid is more tightly coupled to protein. With any membrane protein, the tightly coupled lipid might be specific; that is, the interaction might require that the phospholipid contain specific fatty acid chains or particular polar head groups. There is at present, however, no satisfactory direct evidence for such a distinctive lipid fraction”.*)	The membrane is full of proteins, leaving no membrane fraction unaffected by their presence. Protein–protein interactions have functional important signaling implications. There are lipids in direct contact with the protein (boundary lipids) that provide a special lipid environment for the proteins. Some of these lipids have a fast exchange with bulk lipids (annular lipids), whereas others (non-annular lipids) are tightly bound to certain membrane proteins stabilizing their conformation and/or function.

Proteins interact with the bilayer in two different forms: as *peripheral or extrinsic proteins* (associated to the lipid bilayer polar headgroups) and as *integral or intrinsic proteins* (associated to the hydrophobic matrix).	There are also other proteins that are only part of the time docked to a membrane (*membrane associated proteins*). They are not involved in the microstructure of the membrane; however, they have important membrane functions and dynamics. For example, protein kinases C and annexins (Bazzi and Nelsestuen, 1996).

The membranes are *fluid*. Lipids and proteins have two of three different modes of motion: *rotational* around an axis perpendicular to the plane of the membrane, and freely *translational* along the plane of the membrane. *Transbilayer* diffusion is forbidden because of the energy barrier presented by the hydrophobic matrix to the polar groups of the lipids and proteins.	The high amount of transmembrane proteins plus peripheral proteins plus protein-protein interactions restricts dramatically the lateral diffusion of proteins. The membranes are seen as “more *mosaic* than *fluid.”*^1^ Membrane lipids can also undergo fast transbilayer diffusion (*flip-flop* movements), which can be a protein-helped event or a spontaneous event. Scrambling of lipids contributes to the dynamic transbilayer asymmetry of the membrane; or, contrary to this, to losing the asymmetric condition by triggering a signaling process (i.e., phosphatidylserine flip-flop from the inner to the outer hemilayer and apoptosis).

The two surfaces of membranes are not identical in composition, structure, and distribution of oligosaccharides. This *asymmetry* is based on the forbidden transbilayer diffusion.	Membranes are *asymmetric*. Lipids and proteins are different in each hemilayer, this being a condition that involves lipid transporters or spontaneous lipid movements (Quinn, 2002; Daleke, 2003; van Meer, 2011). Integral proteins are naturally asymmetrical in the membrane after their initial biosynthesis. Asymmetry is essential for cells and its disruption is associated with cell activation or pathological conditions.

The membrane is mainly *homogeneous.* The original model suggested that: *“Such short-range order is probably mediated by specific protein (and perhaps protein-lipid) interactions leading to the formation of stoichiometrically defined aggregates within the membrane. However, in a mosaic membrane with a lipid matrix, the long-range distribution of such aggregates would be expected to be random over the entire surface of the membrane”.*	The bilayer is full of uneven *heterogeneous* patches or domains enriched in certain lipids and proteins, which confer irregular thickness in the membrane. This is the result of certain preference of protein-lipid contacts, mismatch between the length of the hydrophobic transmembrane segments of the proteins and the length of the lipid acyl chains, protein–protein contacts, the anchoring of integral proteins to cytoskeletal proteins, the poor miscibility of certain lipids, etc. These domains have very important functional implications. *Membrane rafts* (Simons and Ikonen, 1997) are one type of membrane domains. They are small (10–200 nm), transient and dynamic (short life, ∼200 ms). These domains, which induce lateral order and heterogeneous organization of membranes, are a consequence of the immiscibility of certain lipids of biological membranes, leading to the coexistence of patches with different physical properties and lipid compositions. They also compartmentalize or segregate certain proteins making more efficient a variety of cellular processes. Rafts domains in eukaryotic cell membranes are liquid-ordered domains rich in cholesterol and sphingomyelin. In model membranes, a mixture of lipids that induce a segregation of liquid-ordered (lo) and liquid-disordered phases (ld) is used to study those domains. A *lo phase* is a phase with higher lateral mobility in the bilayer than in a gel phase but with the lipid acyl chains extended and ordered, whereas a *ld phase* is a fluid phase with the acyl chains of the lipids highly disordered and mobile (Simons and van Meer, 1988; Simons and Ikonen, 1997; Brown and London, 2000; London, 2005; Sonnino and Prinetti, 2013).

The membrane is an *isolated system* with no exchange of matter or energy with the environment.	All kind of signals occur in the membrane contacting with the extracellular and intracellular environment, for example molecules reaching and leaving the membrane in response to stimulus (Watson, 2015; Wen et al., 2018).

*^1^Nicolson (2014) said: “I have re-termed the model as the ‘Fluid—Mosaic Membrane Model’ to highlight the important role of mosaic, aggregate and domain structures in membranes and the restraints on lateral mobility of many if not most membrane protein components.”*

Nowadays, a membrane is thought of as an increasingly complex crowded structure of a great variety of *lipid* and *protein* arrangements with lateral and transverse *asymmetry*, variable *patchiness*, variable *thickness*, and higher *protein* occupancy (Engelman, 2005; Nicolson, 2014). It is universally accepted that biological membranes behave as barriers separating two fluid media and avoiding contact with each other. But being a physical barrier is not its only or main function. Many of the biochemical reactions essential for cell life (metabolic and signaling reactions involving G-protein coupled receptors as the rhodopsin or muscarinic receptor and ion channels as nicotinic, histamine, GABA or glutamate receptors among others transmembrane proteins) occur in the cell membranes, making them a truly important agent in almost all cellular physiological and pathological processes. These reactions imply molecular communication, which involves protein–protein and also protein–lipid interactions. Lipid membranes are not just a “sea” where proteins are embedded, as it was initially postulated by Singer and Nicolson. Lipids (including fatty acids, cholesterol, endocannabinoids, arachidonic acid metabolites as prostaglandins, leukotrienes, and epoxyeicosatrienoic acids, etc.) are active molecules with important implications. Lipids such as chol, cardiolipin, PIP2 and glycolipids condition the function of several transmembrane proteins, a fact reflected in the thousands of research papers that report the effect of these lipids on protein functions (Lee, 2003; Barenholz, 2004; Barrera et al., 2013). Here, we will discuss the implications of lipid-protein interactions at the cell membrane level in AD.

## Crosstalk of Alzheimer’s Disease Pathogenesis and Lipid Membrane

Alzheimer’s disease is the most prevalent neurodegenerative disorder in the elderly, and is characterized by progressive cognitive decline. The main pathophysiological characteristics include extracellular accumulation of β-amyloid senile plaques and intracellular accumulation of neurofibrillary tangles (hyperphosphorylated microtubule-associated tau protein) (Feng and Wang, 2012; Kumar et al., 2015). A disruption of the cholinergic pathways that contribute to the cognitive impairment of AD patients is described in the cerebral cortex and in the basal forebrain. AD implicates the formation of extracellular insoluble peptides derived from the action of two transmembrane enzymes, a β-secretase (β-site APP-cleaving transmembrane aspartic protease, BACE 1) and a γ-secretase (an imprecise multimeric protein complex), on the membrane-bound APP. Aβ peptides of different lengths, containing 39–42 amino acid residues, are produced. 1–40 Aβ is produced more frequently while 1–42 Aβ is the predominant species in senile plaques (Iwatsubo et al., 1994). They are amphiphilic peptides with residues 1–28 constituting a hydrophilic domain and residues 29 up to 42 (which correspond to part of the transmembrane domain of APP), a hydrophobic one (Ji et al., 2002). Whereas low concentrations of 1–40 Aβ are related to neurotrophic properties (Yankner et al., 1990; Zou et al., 2002, 2003), 1–42 Aβ, produced in low amounts under physiological conditions, has a much higher tendency to form oligomers, protofibrils and fibrils, which are the ones that constitute AD brain plaques (Jarrett et al., 1993; Gu and Guo, 2013). The structural-activity relation between these assemblies and the differences between 1–40 Aβ and 1–42 Aβ are under continuous investigation and exceed the aim of this review. Alternatively, APP can be cleaved by another membrane enzyme (α-secretase) between amino acids 16 and 17 of the Aβ region, avoiding Aβ peptides generation and producing a neurotrophic and neuroprotective soluble AβPP (sAβPPα) through a non-amyloidogenic pathway (Thornton et al., 2006; Wang et al., 2016). In neurons, amyloidogenic and non-amyloidogenic pathways compete with each other, jumping between neuroprotection and neurodegeneration (Vetrivel and Thinakaran, 2006; Tan and Gleeson, 2019). Furthermore, in normal brains, 1–42 Aβ is produced in low picomolar concentrations and, as it will be explained later, these low, non-toxic concentrations have physiological implications in synaptic plasticity and memory, among others (Plant et al., 2003; Puzzo et al., 2008, 2011, 2015). In fact, physiological 1–42 Aβ binds to several target molecules as apoE, the receptor for advanced glycosylation end products (RAGE), serpin–enzyme complex receptor (SEC-R) and nicotinic acetylcholine (nACh) receptors (Turner et al., 2003). Thus, although during a person’s lifetime there is a continuous formation of all these peptides, the deregulation of the enzymatic equilibrium with the consequent accumulation of insoluble peptides is characteristic of AD. 1–42 Aβ is the most hydrophobic peptide that forms soluble oligomeric intermediates before aggregating as insoluble plaques with cytotoxic properties in the AD brains. It induces iron and cooper reduction in the brain triggering oxidative stress and damage, it causes calcium homeostasis deregulation probably through lipid perturbation at the cell membrane, and it causes oxy-radicals formation and finally neurodegeneration (Butterfield et al., 2013; Fonseca et al., 2015; Cheignon et al., 2018). Amyloidogenic and non-amyloidogenic pathways are thought to occur in different cellular compartments depending on secretases localization. γ-secretase complex is present in multiple compartments: near 6% in the plasma membrane and the rest in intracellular organelles such as endoplasmic reticulum, late Golgi/*trans*-Golgi network and endosomes (Vetrivel et al., 2004; Chyung et al., 2005). However, α- and β-secretases are more compartmentalized. α-cleavage occurs at the cell surface (Parvathy et al., 1999; Haass et al., 2012; Sun and Roy, 2018). APP is released to the plasma membrane through the secretory pathway and stays there for a short time. Therefore, during this short time, APP is proteolytically processed by α-secretase. Anyway, near 70% of APP is internalized by endocytosis. A fraction of this APP is recycled to the cell surface and another one is degraded in lysosomes. BACE 1 is localized late in the Golgi/*trans*-Golgi network and endosomes and cleaves APP during the endocytic/recycling cycle (Koo and Squazzo, 1994); thus, β-cleavage depends on endocytosis (Koo and Squazzo, 1994; Perez et al., 1999; Huse et al., 2000; Daugherty and Green, 2001; Kamal et al., 2001; Ehehalt et al., 2003) and Aβ is produced mainly in the *trans*-Golgi network during the recycling pathway (Vetrivel and Thinakaran, 2006). Additionally, it was suggested that 1–42 Aβ is produced mainly in the endoplasmatic reticulum whereas 1–40 Aβ is produced in the *trans*-Golgi network (Annaert et al., 1999; Greenfield et al., 1999).

APP cleavage by secretases always happens in a membrane, independently of the subcellular compartment. To understand the importance of this fact, it is recommended to read the general commentary by Lukiw (2013) in *Frontiers in Physiology* titled “Alzheimer’s disease (AD) as a disorder of the plasma membrane,” whereas the author pointed out the implication that the membrane has in the physiopathology of this disease. Several studies postulated that membrane components condition the APP enzymatic processing. Particularly, Chol is a key element in the membrane and it has been related to AD in several ways. Lahdo and De La Fournière-Bessueille (2004) studied the minimum lipid requirements of a monolayer for the insertion of APP. They concluded that APP insertion depends on the Chol content, the Chol/PC and the Chol/SM ratios, and the monolayer membrane order. They identified a critical inflection point at near 30% Chol: at a lower ratio APP localizes in the membrane surface mainly in a β-sheet conformation, whereas as this Chol percentage increases, APP can insert spontaneously into the membrane changing its conformation (Ji et al., 2002; Lahdo and De La Fournière-Bessueille, 2004). Consequently, once APP is confined to the interior of the membrane it can perturb the biophysical properties of this membrane and the activity of several transmembrane or associated-membrane proteins. The Chol concentration and Chol location in brain plasma membranes change throughout a person’s life. At early ages, about 87% of the Chol is localized mainly in the inner layer of the brain plasma membrane, but during aging, the percentage of Chol increases in the outer layer losing the initial transmembrane asymmetry and reaching at least 30 mol%, the critical value with respect to APP membrane insertion (Igbavboa et al., 1996; Wood et al., 2002). In another work, it was suggested that modifications of Chol compartmentation and the equilibrium free cholesterol/cholesteryl esters through acyl-coenzyme A:Chol acyltransferase (ACAT) activation, instead of variations of total membrane Chol, are the determinant of Aβ accumulation and cell dysfunction (Puglielli et al., 2001).

**FIGURE 1:**
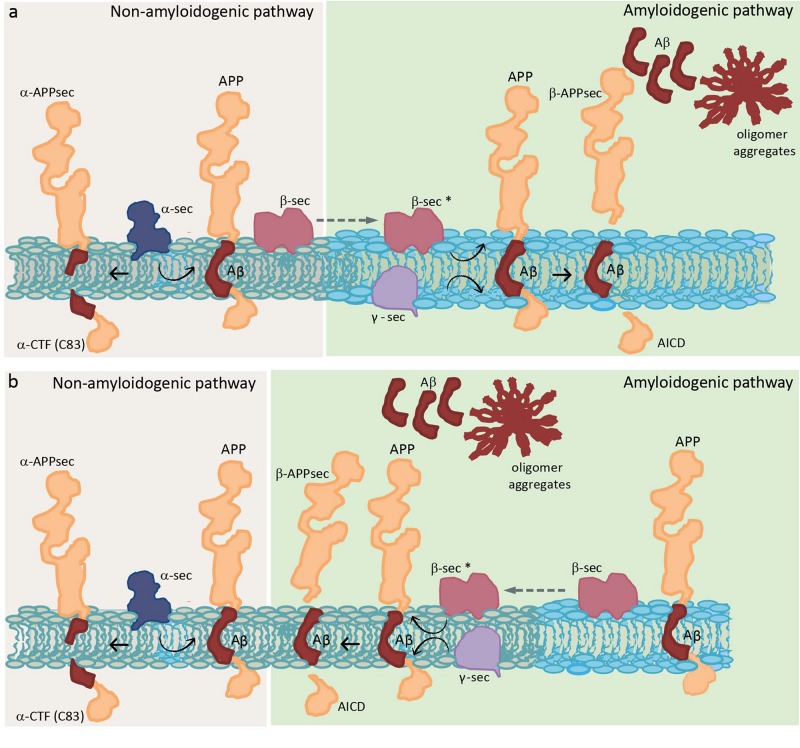
Schematic diagram showing two distinct hypotheses of APP processing, which differ in the membrane location of the whole process. Two different colors are used to represent a raft domain and a liquid-disordered domain (light blue and gray, respectively). **(a)** Hypothesis where β sec is present in both raft and non-raft domains but needs to be in raft domains to be functional (represented as β sec^*^) (Ehehalt et al., 2003). **(b)** Hypothesis where β sec in raft domains corresponds to an inactive pool that needs to relocate to non-raft domains to be functional (Abad-Rodriguez et al., 2004). APP, amyloid precursor protein; α-CTF, C-terminal fragment obtained by α-secretase; α-APPsec, soluble N-terminal APP fragment obtained by α-secretase; Aβ, amyloid β peptide; β-APPsec, soluble N-terminal APP fragment obtained by β-secretase; AICD, APP intracellular domain obtained by the action of γ-secretase on β-CTF or C99 (intermediate peptide that is not shown and corresponds to Aβ plus AICD, obtained in the first step by the action of β-secretase); α-sec, α-secretase; β-sec, β-secretase; and γ-sec, γ-secretase.

The importance of the amount of Chol for APP insertion leads us to think that APP would probably prefer raft domains (Cordy et al., 2006; Reid et al., 2007). Initial studies in the brain showed that both APP and Aβ reside in detergent-insoluble glycolipid-enriched membrane domains (DIG) (Lee et al., 1998), suggesting that those domains are the place in the membrane where the APP processing occurs. The non-amyloidogenic pathway through α-secretase is thought to occur in non-raft domains (Kojro et al., 2001; Reid et al., 2007). On the other hand, although there is no consensus about the localization of APP and BACE 1, there is agreement that APP cleavage by β- and γ-secretase occurs in raft domains (see Table 1 for a detailed explanation of raft domains). Experiments in culture cells showed that overexpressed APP and both secretases enzymes localize in Chol-rich domains (Burns and Duff, 2002; Ehehalt et al., 2003), and that Chol depletion by Chol synthesis inhibition or Chol membrane extraction resulted in a reduction of Aβ production (Simons et al., 1998; Fassbender et al., 2001; Ehehalt et al., 2003). Several studies suggest that APP is present in two cellular pools: one in raft domains and another in non-raft domains. Ehehalt et al. (2003) concluded that this APP membrane compartmentation explains how the same protein could be processed in two different ways (generating Aβ in raft domains and being cleaved by α-secretase in non-raft domains). Furthermore, they said that although BACE 1 is present in both raft and non-raft domains it needs to be in raft domains to be functional, outside these domains the enzyme is inactive (Figure 1a). That is the reason why, when Chol diminishes, Aβ production also diminishes but increases αCTF (C-terminal fragment) or C83, which is a direct product of α-secretase. Thus, Chol regulates the access of α or β secretase to APP (Ehehalt et al., 2003). On the other hand, immediately after this study, a study in human hippocampal membranes showed that the vast majority of APP is located in non-raft domains, while β-secretase BACE 1 is found in two cellular pools: one in raft domains and another in non-raft domains (Abad-Rodriguez et al., 2004). These authors gave an explanation opposite to the previous one: when Chol diminishes, which is what happens in the membrane from AD patients (Mason et al., 1992; Roth et al., 1995), BACE 1 increases in non-raft domains and then an enhancement of amyloid peptide production occurs. They concluded that BACE 1 in raft domains corresponds to an inactive pool that needs to relocate to non-raft domains to perform its activity, and that it is the Chol which directly conditions APP processing by “allowing” BACE 1 to exit or not from neighboring domains (Figure 1b). However, they distinguished between a mild membrane Chol reduction (less than 25%), which results in an increase of APP processing, and a drastic membrane Chol reduction (more than 35%), where an overall disruption of membrane integrity occurs concomitantly with a lower Aβ production. Working with primary cultures of rat hippocampal neurons infected with recombinant Semliki Forest virus (SFV) carrying APP, Simons et al. (1998) arrived to a different conclusion. They showed that depletion of Chol up to 60–70% did not affect the amount of APPsec (the main processed form of APP in neurons obtained by direct α-cleavage), but drastically decreased the amount of Aβ. Therefore, Chol depletion appears to redirect the APP processing from amyloidogenic processing to non-amyloidogenic cleavage. One possible explanation for this is that the small raft-resident pool of APP and BACE 1 is the active one and that it generates C99 to be processed by γ-secretase (Rushworth and Hooper, 2011). Another explanation considers that the amount of both proteins in rafts is so small that the APP processing by BACE 1 is effective once a clustering of raft domains occurs during endocytosis, meanwhile, in the plasma membrane, APP will be mainly cleaved by α-secretase through a non-amyloidogenesis pathway (Ehehalt et al., 2003). Thus, it is possible that APP processing can be altered by membrane lipid composition perturbations. Eckert et al. (2003) showed that Chol depletion decreases the amount of APP in raft domains and, consequently, the production of Aβ. On the other side, Chol increment as in Niemann Pick type C model cells, causes an APP augmentation in raft domains.

A controversial point is where are secretases located, especially β-secretase, and where they function in the membrane. With respect to γ-secretase, however, there is broad consensus. It is postulated that this enzyme is localized in raft domains confirming that the last step in the generation of Aβ occurs in those domains (Vetrivel et al., 2005). These authors postulated that once APP is cleaved by β-secretase, the CTFs (or C99) produced are recruited or sequestered into raft domains where cleavage by γ-secretase takes place. They indicated that ∼20% of BACE 1, less than 5% of APP and more than 70% of CTFs reside in raft domains; and, based on previous work, they assume that all cleavage occurs in these rigid domains (Vetrivel et al., 2005).

By magnetic nuclear resonance of C99, Beel et al. (2010) identified a short sequence of 5 amino acids (VGSNK) between the extracellular segment and the transmembrane domain that interacts with Chol, probably through hydrogen bonds. These authors recognize that although C99 is in raft domains, APP, which has the same loop, localizes mainly in non-raft domains, concluding that one possibility is that APP and C99 have different affinities for Chol. This Chol interaction site is also present in Aβ peptides, thus explaining the reported Chol-Aβ peptides interactions that trigger oligomerization, fibrillization, etc. (Beel et al., 2010) which will be discussed below.

Chol is not only crucial for APP processing in the membrane by compartmentalizing the location of both APP and secretases, but also for modulating the secretases activity. Briefly, Chol positively modulates BACE 1 and γ-secretase activities, and negatively modulates α-secretase (Bodovitz and Klein, 1996; Simons et al., 1998; Frears et al., 1999; Kojro et al., 2001; Wahrle et al., 2002; Ghribi et al., 2006). In lysates from human brain and in cultured cells, a certain amount of Chol stimulated β and γ-secretase activities, but at 20 μM Chol γ-secretase activity was inhibited. It is probable that high Chol can directly stabilize the activities of the enzymes to the maximum level in the correct lipid domain or can reduce enzymes degradation increasing Aβ production (Xiong et al., 2008). Furthermore, APP processing can be modulated by Chol conditioning membrane biophysical properties (Kojro et al., 2001; Fukaya et al., 2007; Kogel et al., 2008; Peters et al., 2009; Yang et al., 2010; Askarova et al., 2011). For example, substitution of Chol by lanosterol or polyunsaturated free fatty acids (PUFAs) induced an increment of membrane fluidity, which was related to an enhancement of α-secretase activity (Kojro et al., 2001; Yang et al., 2010; Askarova et al., 2011).

## Aβ Relationship With and Implications on Cell Membrane

**FIGURE 2:**
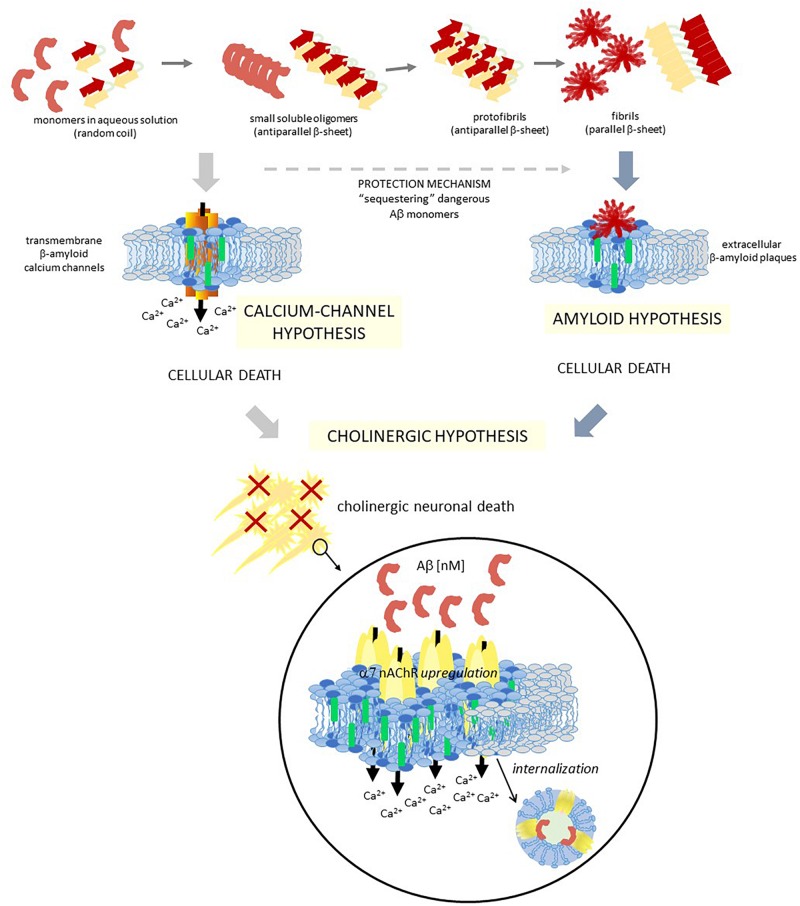
Schematic diagram showing three different hypotheses of AD, which are closely related. Two different colors are used to represent a raft domain and a liquid-disordered domain (light blue and gray, respectively), and also within raft domains, two central lipids are identified for these hypotheses with different colors: chol and GM1 (green and blue, respectively). Aβ, amyloid β peptide; α7 nAChR, α7 nicotinic acetylcholine receptor.

Chol is also important for Aβ peptide action/effect (Eckert et al., 2003; Wood et al., 2014). Aβ peptide can adopt different conformations: a random-coil conformation in aqueous solution, an antiparallel β-sheet in the core of the amyloid plaques, and an α-helix in membranes containing Chol (Ji et al., 2002). It can exist as monomers, oligomers or as amyloid fibrils (Klein et al., 2004; Reid et al., 2007). Several studies indicate that Chol directly binds to APP as was described above (Beel et al., 2010), and also to C99 and monomeric Aβ peptides (Barrett et al., 2012), to oligomers (Ashley et al., 2006), to aggregates (Avdulov et al., 1997), and to fibrils (Harris, 2008). Considering that the mechanism by which Aβ produces brain dysfunction in AD patients is still unknown, these evidences turned the view of Aβ peptides pathogenesis from extracellular plaques (the *“amyloid theory”*: extracellular amyloid plaques are the responsible for cell death; Hardy and Higgins, 1992; Haass, 1996; Rushworth and Hooper, 2011; Serrano-Pozo et al., 2011) to Aβ peptides interaction with the plasma membrane (Relini et al., 2014). A more recent explanation indicates that Aβ monomers or small oligomers are responsible of neuronal death rather than amyloid plaques as it was previously thought (Irvine et al., 2008; Shankar et al., 2008). Furthermore, amyloid plaques reduce neuronal death by sequestering the dangerous Aβ monomers/small oligomers (Arbor et al., 2016) (Figure 2). This explanation is contrary to a previous one that considered that Aβ aggregation in β-sheet conformation, which will finally end as neurotoxic fibrils, is reduced by Aβ insertion as α-helix after interaction with Chol-containing membranes (Ji et al., 2002). They showed that both 1–40 Aβ and 1–42 Aβ peptides prefer Chol enriched LDM and that while in healthy humans the amount of the former peptide is more than twice the second one, the progression of 1–42 Aβ deposition runs in parallel with an increase of this peptide in LDM domains (Oshima et al., 2001; Ji et al., 2002). The authors concluded that Chol enrichment would be beneficial for reducing fibrils, a membrane condition opposite to the one found in AD brains that show a drastic decrease of membrane Chol content and hence do not have the needed conditions for Aβ insertion, favoring the dangerous pathway of Aβ aggregation (Ji et al., 2002). In the case of AD patients, isolated brain membranes showed a significant decrease in membrane Chol, disfavoring the insertion of Aβ into the membrane (Mason et al., 1992; Roth et al., 1995). Thus, Aβ remains in the membrane surface with a great tendency to aggregation and, ultimately, to plaque formation (Ji et al., 2002). By confocal laser microscopy and fluorescence anisotropy, it was shown that 1–42 Aβ peptides interact with raft domains and that there is an inverse correlation between Chol content and membrane perturbation (Cecchi et al., 2009). It was further indicated that a Chol increment decreases amyloid-induced membrane perturbations at lipid rafts by altering the physicochemical properties of the domain (Cecchi et al., 2009). Specific interactions that induce changes in the lipid bilayer conducing to membrane disruption were described between lipids and Aβ peptides (Qiang et al., 2014). Different kinds of interactions were proposed in the last few years. Vestergaard et al. (2010) performed studies of Aβ interactions with model biomimetic membranes and showed that immediately after peptide addition, membrane fluctuations/morphological changes occur. They suggested that both Chol levels and lipid composition affect how Aβ oligomers interact with the membrane. X-ray diffraction studies of the interaction between a 25–35 Aβ peptide and anionic membranes enabled the identification of immiscible Chol plaques when more than 30 mol% Chol was added. The peptide interacts with the bilayers sequestering more Chol molecules into the plaques and, hence, decreasing the amount of Chol in the membrane (Dies et al., 2014).

Chol is not the only important lipid in the Aβ-membrane interactions, there is also GM1, which is a resident lipid of raft domains (Lin et al., 2008). High Chol levels facilitate gangliosides clustering, which is postulated to modulate Aβ oligomerization. These clusters interact with Aβ peptides in a concentration dependent manner inducing Aβ aggregation in β-sheet rich structures with a high Aβ/ganglioside relationship (McLaurin et al., 1998; Ariga et al., 2001; Kakio et al., 2001, 2002; Matsuzaki, 2007). The binding of Aβ to a GM1 cluster favors a conformation transition that depends on the protein density of the membrane. At low peptide/lipid ratios a transition from random coil to α-helix conformation occurs, whereas at high peptide/lipid ratios a β-sheet rich structure appears, which ends in fibrils formation (Matsuzaki et al., 2010; Fukunaga et al., 2012). Significant alterations in the lipid composition of raft domains in frontal cortex of AD patients were described (Martín et al., 2010; Kosicek and Hecimovic, 2013; Fabelo et al., 2014). A detailed study of the lipid composition of DRM from temporal and frontal cortex of AD brains indicated that there was an increment of GM1 and GM2 in both areas of the brain studied (Molander-Melin et al., 2005). This difference, which was considered an early event in the progression of AD, was not observed between samples from brains of different ages or gender (Molander-Melin et al., 2005). Other studies agree with an age-dependent high-density GM1 clustering in synaptosomes (Gylys et al., 2007; Yamamoto et al., 2008) and specific Aβ peptides and GM1 complexes in early AD brains. GM1-Aβ interactions (GAβ) were described in the brain and associated with AD pathology (Yanagisawa et al., 1995; Choo-Smith et al., 1997; Yanagisawa and Ihara, 1998; Kakio et al., 2001, 2002; Yamamoto et al., 2004, 2005; Wakabayashi et al., 2005). Thus, GM1 is postulated as the seed for the formation of amyloid fibrils (Staneva et al., 2018), and several studies considered raft platforms as the site where these interactions happen. Sasahara et al. (2013) showed that the interaction and aggregation of the peptide enhances lipid phase separation because of the GM1 relation with Aβ aggregates. A two-step phase was postulated to occur in membranes of AD patients: at early stages the proportion of GM1 in raft domains increases accelerating Aβ plaques formation and triggering a gradual raft disruption and perturbation of the cellular function that involves these membrane domains; at a later stage, there is also a decrement in Chol content which prevents Aβ aggregation and increases neurotoxicity (Molander-Melin et al., 2005). A more detailed study of the mechanism of Aβ interaction with GM1 indicates that Aβ oligomers, which have increased hydrophobicity compared to Aβ monomers, primarily bind to GM1 initiating progressive alterations such as membrane biophysical and ion permeability perturbations that end in the well-known synaptotoxic effects of Aβ (Hong et al., 2014).

Although all data points to a direct implication of gangliosides on Aβ oligomerization, a ganglioside-independent Aβ oligomerization mechanism was also observed, suggesting that other lipid components or carbohydrates in raft domains would be also implicated (Kim et al., 2006).

One important consequence of these raft domains/Aβ peptide interaction is the occurrence of Aβ peptide aggregation and Ca^+2^ channels formation in raft domains (Lin et al., 2001). In hippocampal cell membranes this process was related to neurotoxicity (Sepúlveda et al., 2014). Di Scala et al. (2013) identified a Chol binding domain in a 20–35 fragment of 1–42 Aβ, which is also present in other peptides with high Chol affinity. Interestingly, although both APP and 1–42 Aβ interact with Chol, they have distinct binding domains [17–40 Aβ for APP (Barrett et al., 2012) and 20–35 Aβ for 1–42 Aβ (Di Scala et al., 2013; Fantini et al., 2014)]. By physicochemical and *in silico* experiments, it was demonstrated that this 20–35 Aβ domain forms oligomeric Ca^2+^ channels in the plasma membrane in a Chol dependent manner (Di Scala et al., 2014). The high interaction with Chol of this 20–35 Aβ domain triggers the helix to an adequate tilted orientation inside the membrane, which allows accurate peptide-peptide interactions and the formation of the circular channel. This oligomeric channel is formed by eight 20–35 Aβ subunits and eight Chol molecules, with a pore size and an external diameter of 1.46 and 4.4 nm, respectively. The formation of these channels could help explain the neurotoxic properties of 1–42 Aβ (Figure 2). Similar *in silico* studies performed with 1–40 Aβ showed that the interactions between Chol and peptide are different to those observed with 1–42 Aβ (Di Scala et al., 2013, 2014). Since the initial proposal of the existence of transmembrane ion channels formed by Aβ peptides (Arispe et al., 1993a,b), lots of studies deepened in the “*β-amyloid calcium channel hypothesis*” (Pollard et al., 1995; Kagan et al., 2002; Kawahara, 2010). The first step for this ion channel formation must be the contact between the peptides and the membrane. It was demonstrated that both the lipid composition of the outer membrane and the structural conformation of the Aβ peptide are crucial for this interaction. In solution, it was possible to find Aβ as β-sheet, α-helix or random coil conformations, being the conformational balance dependent on its concentration. It is postulated that the presence of certain lipids can shift the equilibrium to one preferred conformation. Particularly, it was demonstrated that negatively charged lipids take contact with the peptide by specific electrostatic interactions (Hertel et al., 1997; McLaurin and Chakrabartty, 1997; Terzi et al., 1997). Aβ selectively recognizes and accumulates on GM1-rich membrane domains (Yanagisawa et al., 1995; Wakabayashi et al., 2005; Yanagisawa, 2005), and Aβ insertion into the membrane is critically dependent on the Chol/phospholipids ratio (Ji et al., 2002), as it was detailed above. More recent works showed that the formation of Ca^2+^ pores (“annular protofibrils,” Lashuel et al., 2002) in the plasma membrane is a mechanism dependent on both gangliosides and Chol. As it was described above, amyloid monomers or soluble oligomers interact with a ganglioside at the cell surface, with a specificity that responds to a ganglioside-binding domain for each amyloid protein (common amino acid residues at specific locations, with specific variations for each ganglioside), being the Ca^2+^ pores significantly diminished in ganglioside deprived cells (Di Scala et al., 2016). Based on this “calcium-channel hypothesis” of the AD, a chimeric peptide formed with a minimal ganglioside-binding domain of α-synuclein and two contiguous His residues as in 1–42 Aβ (Yahi and Fantini, 2014) avoid pore formation by 1–42 Aβ. Treatment of WT 5XFAD mice with a sialic-specific lectin (LFA, Limax flavus agglutinin) significantly reduced amyloid depositions in the brain, probably by interfering with the binding of amyloid peptides to gangliosides (Dukhinova et al., 2019). Furthermore, Cascella et al. (2017) showed that different oligomer conformations can perturb Ca^2+^ cell-permeation by both a channel-independent mechanism as annular protofibrils, or by a channel-dependent one (through NMDA-R and AMPA-R).

Aβ peptides that stay in the membrane surface are in a β-sheet conformation, and once inside the membrane they turn their conformation to an α-helix (Yu and Zheng, 2012). Other studies suggested that 1–40 Aβ interacts with the membrane in two sequential steps. The first one involves the formation of a pore-like structure and membrane permeation, and the second one involves subsequent growth of aggregates with fibril formation and lipid clustering around the fiber which implies lipid extraction, membrane fragmentation, and loss of membrane integrity (Engel et al., 2008; Stefani, 2010; Milanesi et al., 2012; Sciacca et al., 2012; Relini et al., 2013; Kotler et al., 2014).

Recently, Rondelli et al. (2016) described more in detail the interactions between cell membranes and Aβ peptides. Those interactions depend on peptide conformation: structural oligomers are imbibed in the outer hemilayer of the membrane triggering more Aβ addition and further elongation; on the other hand, early labile oligomers in equilibrium with monomers are incorporated as monomers deeply in the membrane coming up to the inner hemilayer, whereas Aβ organization leads to pore formation.

A study of the changes induced by 1–42 Aβ on the morphology and the mechanical stability of model membranes with different Chol content indicates that Chol drives 1–42 Aβ toward rafts domains and that at high Chol concentration the presence of the amyloid peptide did not alter any membrane property, thus assigning a protective effect against membrane destabilization by 1–42 Aβ to the presence of Chol (Seghezza et al., 2014). Recently, Staneva et al. (2018) deepened this idea. They observed that 1–42 Aβ has a higher affinity for liquid-disordered (l_d_) than ordered (l_o_) phases, confirming previous results (Ahyayauch et al., 2012). They concluded that the fraction of Aβ in l_o_ domains, probably the functionally important one, might be smaller. While in a l_o_ phase 1–42 Aβ induces practically no changes in the lipid packing, a significant perturbation of the lipid packing by its presence was observed in a l_d_ phase. They focus on the presence of GM1 as a crucial lipid. In l_d_ phases without GM1, the peptide penetrates and messes up the neighboring lipids. However, in the presence of GM1 the peptide interacts with the headgroup of several GM1 promoting a condensing effect and an increased lipid packing and decreases Aβ penetration. The presence of GM1 could affect the line tension between l_o_ and l_d_ domains which in turn affects the kinetics of domains formation, growth, shape and size. Thus, although it cannot be discarded that the functional peptide, or at least a minority of it, binds directly to l_o_ domains, the authors suggested that the fibrillation of Aβ peptides in raft domains is the consequence of a reorganization modulated through Aβ peptides in non-raft domains (Staneva et al., 2018).

Not only specific lipid raft characteristics are necessary for Aβ insertion into the membrane, but also its insertion has consequences on the membrane (Chang et al., 2017). Several studies analyzed the membrane biophysical perturbations caused by Aβ interaction, which could be considered the first step of its biological effect (Kanfer et al., 1999; Chochina et al., 2001; Eckert et al., 2003). A decrease in the fluidity of mouse brain membranes, human lymphocyte membranes and membranes from rat cortex, hippocampus and striatum was observed in the presence of 25–35 Aβ and 1–40 Aβ, and in all cases the effect was dependent on peptide concentration (Müller et al., 1995). Low concentrations of Aβ significantly perturb membrane fluidity by specifically altering the acyl-chain mobility of brain membranes, an effect dependent on peptide length, with almost no effect at the polar head groups (Müller et al., 2001). Lately, it was observed that monomeric 1–40 Aβ has no effect on membrane fluidity, while oligomeric forms do (Peters et al., 2009). Contrary to these results, by exposition of hippocampal neurons to nanomolar concentrations of Aβ oligomers for 24 h we could not observe changes in membrane fluidity tested with three different fluorescence probes (Uranga et al., 2017). Peters et al. (2009) showed that membrane perturbation by Aβ is a consequence of Aβ complexing with GM1; thus, it is possible that in our experiments the cell membrane did not have the correct GM1/Chol relationship. A previous study of the interaction of 1–42 Aβ with planar bilayers had already demonstrated that the Chol content is directly correlated with Aβ assembly on the membrane surface, that during this process membrane changes occur, and that all this process is governed by lipid bilayer composition (Yip et al., 2002). Thus, membrane lipid environment modulates Aβ production and at the same time Aβ causes a membrane perturbation that positively feedbacks its own production (Peters et al., 2009). Moreover, Aβ insertion into the membrane not only potentiates Aβ production but also unspecifically activates a variety of membrane processes which could eventually end in neuronal cell death (Kanfer et al., 1999). 25–35 Aβ peptide interacts with phospholipids through electrostatic interactions favoring peptide aggregation which causes perturbations at the lipid-water interphase of the membrane (Martínez-Senac et al., 1999). Mass spectroscopy studies showed that Aβ inserts into model membranes containing Chol, but not in the absence of Chol (Ji et al., 2002). This study also indicated that the membrane insertion is initiated by the C-terminus of the Aβ peptide which has the hydrophobic domain.

Brain membranes from middle aged mice were more susceptible to Aβ perturbations than membranes from aged mice; and *in vitro* studies showed that a decrease in membrane Chol content enhanced Aβ effect, while an increase in membrane Chol strongly decreased the perturbation effect (Kirsch et al., 2002), suggesting that Chol protects neuronal membranes from Aβ perturbations and neurotoxicity (Eckert et al., 2003). However, they also observed *in vivo* that a reduction of Chol levels by approx. 30% by treatment with lovastatin (HMG-CoA-reductase inhibitor) resulted in moderated membrane alterations without acyl chain flexibility perturbations and reduced Aβ bulk fluidity perturbation (Kirsch et al., 2002). A possible explanation is that Chol membrane modification involves different membrane Chol pools with different sensitivity for Aβ perturbations whether it is *in vitro* or *in vivo*, with the one at the membrane acyl-chain being the most receptive (Kirsch et al., 2003).

Another important consequence of an enhanced Aβ production linked to lipid membrane is oxidative stress with an excess of lipid peroxidation and increased lipid susceptibility to oxidative damage, which exacerbates Aβ toxicity in the membrane (Behl et al., 1994; Opazo et al., 2002; Cutler et al., 2004; Boyd-Kimball et al., 2005; Wu and Luo, 2005). It is reported that Aβ prefers to interact with membranes with high oxidatively damaged phospholipids (Zampagni et al., 2010), particularly in raft domains, and that these membranes promote misfolding and aggregations of Aβ peptides into fibrils (Shringarpure et al., 2000; Magni et al., 2002; Zhang et al., 2004; Bieschke et al., 2005; Lee et al., 2006; Murray et al., 2007), whereas the misfolded peptides promote more oxidative damage in the membrane, conducing to a positive feedback (Murray et al., 2007). Aβ increases 4-hydroxy-2-nonenal (HNE) production which promotes oxidative damage and also induces Aβ to form β-structure and amyloid fibrils (Mark et al., 1997; Lauderback et al., 2001; Murray et al., 2005, 2007).

Even though it is not a topic of interest for this review, it is important to remember that just as Chol is a crucial lipid molecule for Aβ processing and Aβ membrane effects, the round trip is also valid since Aβ has an impact on Chol homeostasis (Koudinova et al., 2000; Michikawa et al., 2001; Gong et al., 2002; Michikawa, 2003; Koudinov and Koudinova, 2004; Grimm et al., 2005, 2007). This ultimate effect suggests that Aβ down-regulates Chol content and also raft content (Beel et al., 2010). Thus, the peptide behaves as a Chol sensor: when there is high Chol content in a membrane, the amyloidogenic pathway is favored and, thus, an enhancement takes place in Aβ processing, which in turn reduces both Chol uptake and biosynthesis, following up a negative feedback mechanism (Beel et al., 2010).

## Crosstalk Between Amyloid Hypothesis and Cholinergic Hypothesis

The basis of AD pathogenesis is still controversial today, even though several hypotheses try to explain it, such as the Aβ amyloid cascade (Hardy and Allsop, 1991; Hardy and Higgins, 1992), the hyperphosphorylated microtubule-associated tau (Götz et al., 2004), abnormalities of the cholinergic system (Bartus et al., 1982), oxidative stress (Butterfield and Boyd-Kimball, 2005), etc. Even though the amyloid hypothesis is the most popular explanation for the mechanism of AD, it fails to explain several aspects of this multifactorial etiopathology (Herrup, 2015). In addition, until now, the majority of clinical trials conducted to diminish the amount of Aβ did not give good results (Puzzo et al., 2015; Maia and Sousa, 2019). Although these failures are not enough to discard the amyloid hypothesis (see for example Rosenblum, 2014), attention is now focused on the cholinergic hypothesis since it became the main therapeutic strategy for this disease (Figure 2). As we will work out in the following paragraphs, these two hypotheses are highly linked.

The cholinergic system involves two families of receptors, nAChR and muscarinic acetylcholine receptors (mAChR). Although both types of receptors are related with cognitive processes (Ghoneim and Mewaldt, 1977; Petersen, 1977; Sarter and Paolone, 2011) and are affected in AD, only the relation between nAChR and AD has been largely studied (Lombardo and Maskos, 2014).

The nAChR is an integral membrane protein that belongs to the Cys-loop superfamily of ligand-gated ion channels (Karlin and Akabas, 1995; Le Novère and Changeux, 1995; Changeux and Edelstein, 1998; Paterson and Nordberg, 2000). The binding of its natural agonist acetylcholine triggers a conformational change that ends in the opening of a channel and the flux of positive ions across the membrane, causing membrane depolarization and a subsequence intracellular cascade of events (Lindstrom, 2003; Brown, 2006; McKay et al., 2007; Pohanka, 2012). The nAChR presents a pentameric arrangement, with each subunit having a large N-terminal extracellular domain, four transmembrane segments (M1–M4), a small cytoplasmic domain between M3 and M4, and a short C-terminal extracellular domain. To this day, 16 different nAChR subunits (including: α1-7, α9-10, β1-4, γ, δ, and ε) that form homologous and heterologous receptors with distinct structures, functions and locations are known (Champtiaux et al., 2003; Dajas-Bailador and Wonnacott, 2004; Fucile, 2004; Giniatullin et al., 2005; Gotti et al., 2006a, 2007, 2009; Albuquerque et al., 2009; Shen and Yakel, 2009). The muscle-type nAChR of the electric organ of *Torpedo*, first receptor described and still the prototype of the family, is formed by α1_2_β1δγ (similar to embryonic muscle nAChR of vertebrates, which change to α1_2_β1εγ in adult). Two receptor subtypes are highly expressed in the central nervous system: the heteropentamer α4β2 nAChR and the homopentamer α7 nAChR (Schmidt and Freeman, 1980; Sargent et al., 1991; Clarke, 1992; Sargent and Garrett, 1995; Cooper et al., 1999; Nashmi et al., 2003; Scholze et al., 2011). The latter is particularly important in AD (Ma and Qian, 2019). It is present in high density in the striatum, thalamus, neocortex, and limbic system suggesting a central role in normal cognition and, hence, in age-related cognitive decline (Bigl et al., 1982; Mesulam et al., 1983; Muir et al., 1993; Sarter and Bruno, 1997; Wenk, 1997; Woolf, 1998; Guillem et al., 2011). It was shown that α7 nAChR is important for growth, development and aging, regulating the plasticity of the neural circuit, neuronal differentiation, proliferation, apoptosis and clearance of aged neurons (Nees, 2015). The levels of this receptor change during development and adult stage, and in AD patients, they decrease significantly (Bowen et al., 1976; Perry et al., 1981, 1985, 1987, 1988; Whitehouse et al., 1981, 1982, 1986; Coyle et al., 1983; Shimohama et al., 1986; Nordberg et al., 1995; Paterson and Nordberg, 2000; Auld et al., 2002; Gotti et al., 2006b; Kim et al., 2013; Ma and Qian, 2019).

Activation of the α7 nAChR opens a high permeability Ca^++^ channel that consequently activates voltage-dependent Ca^++^ channels (Perry et al., 1992; Sharma and Vijayaraghavan, 2001) and triggers an intracellular signaling cascade through activation of a protein kinase. In the case of activation of presynaptic α7 nAChR, the final event is the fusion of vesicles loaded with neurotransmitters (glutamic acid, norepinephrine, acetylcholine, dopamine and GABA) to the presynaptic membrane and the massive release of these neurotransmitters to the synaptic cleft (Wonnacott et al., 2006; Ma and Qian, 2019). Postsynaptic α7 nAChR depolarize the postsynaptic membrane and participate in signal transduction (Messi et al., 1997; Morley and Happe, 2000; Berg and Conroy, 2002). ACE metabolizes acetylcholine after its release to the synaptic cleft ending the cholinergic stimulus (Bowen et al., 1976; Davies and Maloney, 1976; Coyle et al., 1983; Auld et al., 2002).

The cholinergic hypothesis of AD focuses on the fact that in brains of AD patients there is a decrease in the total amount of nAChRs (Whitehouse et al., 1982; Banerjee et al., 2000), which is an outcome of progressive death of forebrain cholinergic neurons with an extended cholinergic presynaptic denervation (Bartus et al., 1982; Court et al., 2000; Graham et al., 2002; Contestabile, 2011; Hampel et al., 2018). This is considered a consequence of enhanced Aβ production (Liu and Wu, 2006). Banerjee et al. (2000) observed that in the remaining cholinergic neurons there was a higher amount of nAChR, which suggests a possible compensatory mechanism. Many efforts were performed to ameliorate this loss. However, current approved pharmacological agents, such as physostigmine, tacrine, donepezil, rivastigmine and galantamine (Martorana et al., 2010), are targeted to inhibit ACE function increasing the amount of acetylcholine at the synapse cleft and ameliorating the clinical symptoms of AD without halting the progress of the disease.

The affinity of α7 nAChR for 1–42 Aβ is in the low picomolar concentration, a range estimated to occur in healthy brains, while the affinity of α4β2 nAChR for 1–42 Aβ is between 100 to 5000 times lower (Wang et al., 2000a); thus, it is expected that both α7 nAChR and 1–42 Aβ could associate under physiological conditions. Puzzo et al. (2015) hypothesized that under physiological conditions a positive feedback mechanism occurs: synaptic activity induces Aβ release that acts as an endogenous ligand and modulates α7 nAChR, which in turn induces release of neurotransmitters and enhances synaptic plasticity and memory. Under pathological conditions, abnormal accumulation of Aβ (nanomolar concentration, Näslund et al., 1994, 2000; Tapiola et al., 2000; Andreasen et al., 2003) induces a negative feedback mechanism which implies inhibition and internalization of α7 nAChR, leading to synaptic dysfunction and memory loss. The Aβ-α7 complex influences tau hyperphosphorylation (Wang et al., 2003) and its internalization leads to plaque formation (Nagele et al., 2003, 2004; Dineley, 2007).

It is thought that the soluble form of Aβ interacts with α7 nAChR with apparently high affinity (Wang et al., 2000a) regulating its function (Dineley et al., 2001; Liu et al., 2001; Pettit et al., 2001). However, there is no consensus about the nature and consequences of this interaction (Farhat and Ahmed, 2017). While several studies propose an agonist-like effect for presynaptic nicotinic receptors (Dineley et al., 2002; Dougherty et al., 2003; Wu et al., 2007; Puzzo et al., 2008; Mehta et al., 2009; Lilja et al., 2011; Arora et al., 2013), others propose an inhibitory action (Dineley et al., 2001; Liu et al., 2001; Pettit et al., 2001; Tozaki et al., 2002; Lee and Wang, 2003; Wu et al., 2004; Wang et al., 2009; Parri et al., 2011), and others a concentration-dependent relationship with a stimulatory effect at picomolar Aβ concentration and an inhibitory effect at high nanomolar Aβ concentration (Puzzo et al., 2008). The variability between all the performed studies is so large in terms of *in vitro* and *in vivo* models, Aβ concentrations and Aβ preparations/conformations, and other conditions, that it is difficult to find a rule for the data obtained. Khan et al. (2010) gave a possible explanation for these inconsistency centered in a different Aβ effect on pre or postsynaptic receptors. Aβ induces a rapid stabilization of an inactive/desensitized state of postsynaptic receptors, resulting in an antagonist effect, and a slower desensitization of presynaptic receptors resulting in an agonist-like effect. The authors pointed to differences in the lipid microenvironment of the pre and postsynaptic α7 nAChR for these different desensitization rates. Presynaptic terminals have abundance of raft domains, and experimental disruption of these domains dramatically attenuates Aβ evoked α7 nAChR currents (Khan et al., 2010). With respect to the concentration-dependent effect, it is important to take into consideration that in a normal central nervous system 1–42 Aβ is found, although at low picomolar concentrations. Under this condition, it is postulated that Aβ exerts a positive effect on synaptic plasticity and memory formation (Phinney et al., 2003; Plant et al., 2003; Puzzo et al., 2008, 2011, 2015; Puzzo and Arancio, 2013). However, in a pathological condition, Aβ cannot exert its physiological function and hence a feedback mechanism induces more Aβ production, leading to an enhancement of the peptide with the subsequent reduction of α7 nAChR with Aβ removal and synaptic alteration and memory loss (Phinney et al., 2003; Puzzo et al., 2015).

Interaction of Aβ with α7 nAChR increases Aβ internalization (Nagele et al., 2002; D’Andrea and Nagele, 2006) and accumulation in lysosomes causing an excessive intraneuronal 1-42 Aβ accumulation. The majority of the amyloid plaques proceed from the lysis of degenerated, Aβ-overburdened neurons (Wang et al., 2000b; Gyure et al., 2001; Langui et al., 2004; Nunomura et al., 2010; Palop and Mucke, 2010; Li et al., 2011; Deutsch et al., 2014, 2016). Additionally, the formation of the Aβ-α7 nAChR complex may influence the membrane lipid and membrane protein organization (Deutsch et al., 2014, 2016; Ma and Qian, 2019). At the same time, the internalization of the Aβ-α7 nAChR complex triggers an upregulation of the α7 nAChR and magnifies the toxicity of the pathology (Molinari et al., 1998; Xiu et al., 2005; Yu et al., 2005; Liu et al., 2013, 2015; Shen and Wu, 2015) (Figure 2). In AD patients and preclinical AD models, a high expression of α7 nAChR was described (Hellström-Lindahl et al., 1999, 2004a,b; Dineley et al., 2002; Jones et al., 2006; Counts et al., 2007; Ikonomovic et al., 2009). Chronic exposure to Aβ enhances the expression of α7 nAChR in neuron and glia cells (Yu et al., 2005; Liu et al., 2013). Also, an age-dependent increase of cell surface α7 nAChR was observed in 5xFAD mice, a model that rapidly develops amyloid pathology (Jin et al., 2015). Several studies contributed to this hypothesis. Treatment of PC12 cells with 1–42 Aβ increased cell surface α7 nAChR, suggesting that the peptide induces translocation of the receptor toward the plasma membrane (Jin et al., 2015). They observed that the agonist nicotine prevented Aβ induced cell death, whereas the competitive antagonist α-bungarotoxin potentiates the peptide effect, indicating that α7 nAChR plays a role in protecting neuronal cells from Aβ 1–42 peptide (Dziewczapolski et al., 2009; Wang et al., 2009; Jin et al., 2015). Contrary to this, Liu et al. (2015) concluded that upregulation of α7 nAChR induced by Aβ is necessary to mediate peptide neurotoxicity, both in hippocampal neurons and differentiated cholinergic SH-SY5Ycells. α7 nAChR function, which is exacerbated by its upregulation, may be necessary for the toxicity of Aβ aggregates; this effect was prevented by α7 nAChR inhibition or deletion. Previous studies showed that the blockade of α7 nAChR significantly ameliorated attentional deficits (Levin et al., 2013; Burke et al., 2014). Likewise, the deletion of α7 nAChR gene was correlated with an improvement in synaptic plasticity and a reduction in cognitive deficiency (Dziewczapolski et al., 2009). Two possible cytotoxic α7 nAChR-mediated mechanisms were proposed: one considers that the α7 nAChR increment in the membrane conduces to a high calcium permeability, which could be the ultimate responsible for cell toxicity, and the other that the high Aβ-α7 nAChR complex internalization and intracellular accumulation leads to neurotoxicity (Liu et al., 2015). Thus, while several studies point to α7 nAChR activation as a beneficial treatment, others suggest that a function inhibition for a beneficial effect is necessary.

A different hypothesis about Aβ and α7 nAChR relationship was postulated by Small et al. (2007). They concluded that Aβ does not bind directly to α7 nAChR but to the lipids of the plasma membrane, and that the perturbation of the structure or fluidity of the lipid microenvironment of the receptor could be the responsible for toxicity through an alteration of the receptor function. Their conclusion is supported by previous evidence that showed that Aβ binds strongly to lipids (Subasinghe et al., 2003; Hou et al., 2005). We will return to this issue later.

We here described the most relevant information about the interaction between Aβ and α7 nAChR, and its final consequences, focusing on the events that occur through the membrane. However, not only the interactions between Aβ and α7 nAChR are important. Other proteins that interact with α7 nAChR including Lynx proteins, NMDA-receptors and the Wnt/β-catenin pathway are important as well. All those interactions that modulate receptor function are specifically altered in AD and can lead to differences in the clinical effect of nAChR ligands in AD (Thomsen et al., 2016). It is also important to take into account that there is an internal cascade of signaling downstream α7 nAChR activation that involves several other active molecules, such as glycogen synthase kinase-3β (GSK-3β), phosphoinosite 3-kinase (PI3K)-Akt, Wnt and the mitogen-activated protein kinase (MAPK) signaling pathway, which are also altered in AD (see Ma and Qian, 2019 for a further explanation).

The last step in cholinergic signaling is the degradation of acetylcholine by the enzyme ACE to end the synaptic transmission. ACE is a globular non-transmembrane protein that can exist in different molecular forms, depending on the splicing of the ACE gene (Henderson et al., 2010). Although all ACE molecular forms and variants have similar catalytic activity, they also have other non-catalytic, non-classical functions, which depend on the multiple molecular forms of this enzyme and on cell types and cellular compartments (Small et al., 1996; Grisaru et al., 1999; Massoulié, 2002; Hicks et al., 2011). In non-neuronal tissues, ACE regulates cell proliferation, differentiation, apoptosis and cell–cell interaction, which is important to take into consideration when ACE inhibitors for AD are designed (Lazarevic-Pasti et al., 2017). ACE_T_ is the predominant form in central nervous system, which has a C-terminal α-helix peptide of 40 amino acids named T peptide. Through disulphure bondings between these peptides they can be found as homodimers and homotetramers of ACE_T_. Also, the T peptide binds to hydrophobic proline-rich domains of membrane anchoring-proteins (like collagen-like Q subunit in NMJ and proline-rich membrane anchor, PRiMA, in the central nervous system; Massoulié et al., 2005). In the central nervous system, the majority of ACE is found as tetrameric ACE_T_ (G4) bound to PRiMA (Navaratnam et al., 2000; Perrier et al., 2002; Massoulié et al., 2005), which constitute the functional units at cholinergic synapse (Perrier et al., 2002; Dvir et al., 2010; Henderson et al., 2010; Hicks et al., 2011). PRiMA could bring the membrane-bound ACE together with other proteins in specialized membrane areas, such as raft domains, specifically with α7 nAChR at basal forebrains cholinergic neurons (Henderson et al., 2005; Hicks et al., 2012). A significant proportion of ACE_T_ is effectively located in raft domains through a Chol-binding domain of 13 amino acids of PRiMA (a CRAC, Chol recognition amino acid consensus, sequence), and Chol depletion or mutations at this domain reduced the lipid raft-PRiMA association (Xie et al., 2010a,b). A diminution of ACE activity in the cerebral cortex and other areas in AD patients was described, being the G4-PRiMA complex the ACE form markedly altered, whereas the ACE monomeric form was almost preserved (Atack et al., 1983; Fishman et al., 1986; Sáez-Valero et al., 1999). Interactions of PRiMA subunit with presenilin 1 (PS1, the catalytic subunit of γ-secretase), which is an aspartyl protease that cleaves substrates inside membrane, were described to occur in raft domains (García-Ayllón et al., 2014). This interaction could explain, in part, the cellular release of ACE through a shedding mechanism that was postulated to involve a metalloprotease (Hicks et al., 2013a). Furthermore, a direct relationship between PS1 and ACE was observed, with an overexpression of ACE related to higher levels of PS1, ACE knockdown leaded to decreased PS1 and a mutated PS1 was related with decreased ACE in the brain (Silveyra et al., 2008, 2012). At the same time, it was also observed that ACE inhibits AβPP processing through γ-secretase (Niu et al., 2012), perhaps, acting as an inhibitor of the secretase by interacting with PS1 (Campanari et al., 2014). In AD, ACE activity is diminished and hence impedes its potentiality to modulate γ-secretase (Campanari et al., 2014).

Interactions of ACE with Aβ are important in AD (Inestrosa et al., 1996; Wang et al., 2000b; Small et al., 2007), as the peptide alters several ACE properties such as its pH optimum and inhibitor sensitivity (Geula and Mesulam, 1989), making Aβ even more neurotoxic (Inestrosa et al., 1996; Alvarez et al., 1998). Moreover, ACE was detected in amyloid plaques evidencing the high affinity between both molecules and suggesting that ACE could promote Aβ aggregation (Morán et al., 1993; Inestrosa et al., 1996). Even more, in some cerebral areas of AD patients almost all ACE is in these complexes. The binding between Aβ and ACE occurs at the ACE peripheral anionic site (PAS); ACE inhibitors that bind to the anionic site (i.e., propidium), as well as antibodies against it, significantly reduce fibril formation (Reyes et al., 1997; Bartolini et al., 2003). Although the ACE catalytic domain does not participate of this interaction (Inestrosa et al., 1996), new compounds with a dual action (blocking PAS and catalytic site) are being designed, looking for the prevention of fibril aggregation with the aim of reversing the progression of the disease and, at the same time, inhibiting acetylcholine degradation to ameliorate the symptomatology (Alptüzün et al., 2010).

Furthermore, a negative relationship between APP and ACE was observed, as an overexpression of APP repressed ACE transcription with reductions of both ACE levels and ACE activity (Hicks et al., 2013b). A similar negative regulation was observed between APP and PRiMA; however, it is not clear if there is a direct downregulation by APP or if this diminution is a consequence of decreased ACE levels (Hicks et al., 2013b; Nalivaeva and Turner, 2016). The authors proposed that this ACE downregulation could be a novel neuroprotective function of APP.

## nAchR and Membrane Lipids

As we said in the previous section, there are several nAChR subtypes depending on the individual pentameric arrangement. Summing up, all nAChR have two well defined structural domains: the neurotransmitter-binding site extracellular domain and the transmembrane domain containing the ion pore. Whereas the extracellular domain is the site where the agonists or different activators/inhibitors bind, the transmembrane region, besides having the ion pore, exhibits extensive contacts with the surrounding lipids through structural motifs remarkably conserved along phylogenic evolution (Antollini et al., 2005; Unwin, 2005; Jha et al., 2007; Baenziger and Corringer, 2011; Baenziger and daCosta, 2013; Barrantes, 2015). It is well known that a correct allosteric coupling between both domains is crucial for nAChR function, strongly dependent on its surrounding lipid, which modulates the relative proportion of nAChR in its resting or desensitized states (daCosta et al., 2002; Baenziger et al., 2000, 2008, 2015; daCosta and Baenziger, 2009; Barrantes, 2010; Barrantes et al., 2010; Hénault et al., 2015). The most studied nicotinic receptor is the muscle nAChR, which is not only the paradigm of all other nAChR but also of the entire cys-loop superfamily. In the following paragraphs we will discuss the relationship between distinct lipids or raft domains and the muscle nAChR, knowledge that can be extended to other members of the family, in particular to α7 nAChR.

Several years ago, Marsh and Barrantes (1978) described a layer of immobilized lipids that encircle the muscle nAChR with characteristics different from those of bulk lipids. Subsequent studies assigned an important role to these bounded lipids on muscle nAChR (Criado et al., 1982; Ellena et al., 1983; Ochoa et al., 1983; Sunshine and McNamee, 1992, 1994; Narayanaswami et al., 1993; Fernández-Ballester et al., 1994; Dreger et al., 1997; Barrantes, 2002, 2007; Quesada et al., 2016). The presence of both Chol and negatively charged lipids in the nAChR-lipid microenvironment is necessary to stabilize the nAChR in a functional conformation (Criado et al., 1984; Fong and McNamee, 1986; Butler and McNamee, 1993; Méthot et al., 1995; Antollini et al., 1996). However, there is no consensus about if it is the entity/identity of the lipid itself or the fluidity that each lipid confers to the membrane the responsible of this role. In spite of this controversy, the importance of a proper lipid microenvironment for muscle nAChR becomes clear when highly hydrophobic molecules, such as free fatty acids or steroids, perturb nAChR function through the membrane localizing at the lipid-nAChR interphase (Andreasen and McNamee, 1980; Villar et al., 1988; Bouzat et al., 1993; Bouzat and Barrantes, 1996; Nurowska and Ruzzier, 1996, 2002; Minota and Watanabe, 1997; Blanton et al., 1999; Garbus et al., 2001, 2002; Antollini and Barrantes, 2002, 2016; Fernández Nievas et al., 2007, 2008). Working with reconstituted *Torpedo* nAChR, Jones and McNamee (1988) distinguished two different populations of lipids in the nAChR-lipid microenvironment region: annular and non-annular lipids. Annular lipids interact with the protein in a relatively less specific manner with a fast rate of exchange with bulk lipids. Contrarily, non-annular lipids are in close contact with the protein, probably in between α-helix transmembrane segments or subunits, and can be associated to lipid binding sites with a slow exchange rate with bulk lipids (Lee, 2003). We identified the same two types of lipids in native *Torpedo* membranes (Antollini and Barrantes, 1998). The entity/identity of non-annular lipids are considered crucial for nAChR function; in the case of annular lipids the biophysical characteristics are more relevant. This is in concordance with other studies that assigned several roles to the lipids in a membrane, two of the main ones being: a collective one, in which they form a viscoelastic lipid “solvent” with the above-mentioned heterogeneities; and an individual one as signaling molecules (Piomelli et al., 2007).

Two annular lipids that are of particular interest are negative lipids and SM. With respect to the requirement of negative lipids, PA is particularly of interest. The segregation of PA domains containing nAChR and the stabilization of a functional conformation of the receptor by PA were described (daCosta et al., 2002, 2004; Poveda et al., 2002, 2008; Wenz and Barrantes, 2005; Dickey and Faller, 2008). SM showed moderated affinity for the nAChR (Bonini et al., 2002) but it is important for proper nAChR stability in the membrane. Its deficit affects the efficiency of the nAChR assembly process and the nAChR targeting to the membrane and increases the rate of turnover (Roccamo et al., 1999; Baier and Barrantes, 2007). Moreover, SM is important for membrane biophysical properties as it is asymmetrically distributed between both membrane hemilayers and it is one of the main actors of lipid raft domains, being both aspects that impact on nAChR (Perillo et al., 2016).

A separate paragraph is for Chol, a key lipid for nAChR (Middlemas and Raftery, 1987). This lipid molecule can be found in every region of a membrane: as a bulk, annular or non-annular lipid. In the first two cases, it probably plays an important function conditioning the physical properties of the environment, mainly because of its participation in raft domain formation and in the maintenance of the asymmetrical membrane condition. As a non-annular lipid, the occurrence of allosteric binding sites is postulated (Addona et al., 1998). It was suggested that the binding domain for Chol is at the nAChR lipid-protein interface, taking contact with the transmembrane subunits αM4, αM1, and γM4 (Corbin et al., 1998); other studies identified interactions of Chol with the transmembrane segments M1, M3, and M4 of each subunit (Hamouda et al., 2006). By fluorescence quenching and energy-transfer measurements of *T. californica* reconstituted membranes, sites accessible to Chol but not to phospholipids were identified (Narayanaswami and McNamee, 1993). Using Molecular Dynamics simulations of the nAChR structure, Brannigan et al. (2008) identified 15 Chol binding sites, large hydrophobic intersubunit and intrasubunit gaps. The location of Chol molecules at these sites improved nAChR stability; and in the case of intrasubunit sites, occupation of these sites by Chol precludes the nAChR from collapsing. A recent study using coarse-grained molecular dynamics simulations suggested that while long n-3 chains (in this case, docosahexaenoic acid, 22:6) have a high propensity for annular and non-annular sites, displacing Chol and occupying sites even deeper within the bundle, shorter n-6 chains do not displace Chol from non-annular sites as efficiently as long n-3 chains (Sharp et al., 2019).

Considering the intimate and close relationship between Chol and the muscle nAChR, studies looking for a consensus about specific Chol domains in the nAChR subunits were performed. A CRAC sequence in a region immediately adjacent to the M1 transmembrane domain of all the subunits of the muscle nAChR was identified (Baier et al., 2011). These sequences are located exiting the membrane bilayer, which suggests that they are probably not good partners for Chol in the hydrophobic membrane environment. However, a novel Chol recognizing domain was identified by *in silico* studies, a sequence opposite to a CRAC one (inverted CRAC or “CARC” sequence) at M1, M3, and M4, which is located inside the membrane and is highly preserved in the evolutionary scale, from prokaryotes to humans (Baier et al., 2011; Di Scala et al., 2017). These *in silico* results were also experimentally confirmed (Fantini et al., 2016). Furthermore, the authors concluded that a CARC sequence generally exhibits more affinity for Chol than a CRAC one (Fantini and Barrantes, 2013), and that it is of high affinity, lipid specific, and saturable (Fantini et al., 2016).

Chol not only conditions nAChR function but also its stability in the plasma membrane. There are some controversies about how the nAChR is organized in the membrane. At the NMJ, supramolecular aggregations of nAChRs (micron-sized two-dimensional clusters) are postulated to occur in Chol-rich lipid microdomains, together with several postsynaptic proteins including rapsyn, MuSK and Src-family kinases. Chol would stabilize NMJ and promote its maturation (Willmann et al., 2006). Depletion of cell-surface Chol produced a marked alteration of the organization of the nAChR (Kellner et al., 2007). One hypothesis for this situation is that after an agrin (extracellular heparan sulfate proteoglycan that aggregates nAChRs on cultured myotubes) stimulus, nAChR and MuSK translocate into raft domains where nAChR clustering occurs, as raft domains concentrate the agrin/MuSK signaling, nAChR and rapsyn. Disruption of these microdomains by Chol depletion inhibits agrin stimulation and formation and maintenance of nAChR clusters (Zhu et al., 2006). A contemporary study suggested that agrin causes the translocation of nAChR into raft domains, which is in agreement with the mentioned hypothesis (Campagna and Fallon, 2006). A slightly different hypothesis indicates that agrin does not reclute nAChRs into raft domains, as they are already in those domains independently of agrin activation, but it triggers the coalescence of raft domains conducing to nAChR clustering and it is also responsible for its maintenance, as Chol is necessary for all this process (Stetzkowski-Marden et al., 2006a,b; Cartaud et al., 2011). A previous study supports this hypothesis where the authors observed that nAChR subunits and rapsyn are cotargeted in the exocytic pathway to the cell surface inserted in Chol-rich microdomains (Marchand et al., 2002). Furthermore, Chol depletion affects the maintenance of the nAChR in the plasma membrane by several mechanisms. Treatments of cells with methyl-β-cyclodextrin, which extracts Chol from the membrane, enhanced nAChR internalization by endocytosis with a marked decrease of the number of nAChR domains, concomitantly with a gain-of-function of the remaining nAChR (Borroni et al., 2007; Borroni and Barrantes, 2011; Kamerbeek et al., 2013). Furthermore, chronic treatments with mevinolin, an inhibitor of 3-hydroxy-3-methyl-glutaryl-CoA reductase and hence of Chol synthesis, inhibited the trafficking of the receptor toward the membrane surface, which caused low nAChR cell-surface expression, and increased the intracellular nAChR pools (Pediconi et al., 2004). Moreover, Chol conditions muscle nAChR cell-surface diffusion (Baier et al., 2010; Mosqueira et al., 2018) and nAChR stability in confined raft domains (Mosqueira et al., 2018).

Different results of the interaction between muscle nAChR and lipid domains were obtained in model systems. We observed that reconstituted *Torpedo* nAChR in symmetric model membranes with coexistence of liquid-ordered (l_o_) and liquid-disordered (l_d_) domains was distributed homogeneously, without preference for any domain (Bermúdez et al., 2010). However, similar experiments with a synthetic peptide corresponding to the γM4 peptide showed a marked preference of this peptide for l_o_ domains (de Almeida et al., 2004; Bermúdez et al., 2010). Thus, although this transmembrane segment could give the nAChR the potentiality to localize in raft domains, it is not sufficient and other conditions must occur which influence nAChR partition profile. One of these mentioned conditions is membrane asymmetry. By increasing SM in the outer hemilayer, we observed an increment of the *Torpedo* nAChR in l_o_ domains, and the same was observed when specific SM species instead of brain SM were used in symmetric models (Perillo et al., 2016). Recently, by using coarse-grained molecular dynamics simulations of nAChR inserted in a ternary system of DPPC:Chol:PE or PC with PUFA, the authors concluded that nAChR partitioned in l_d_ domains poor in Chol (Sharp et al., 2019). The simulated membrane, despite having l_o_ and l_d_ domains, (a) did not have SM of any species, which is a critical lipid for raft domains in biological membranes, (b) used PUFA which are known to behave as nAChR inhibitors probably by competition with Chol for non-annular sites, as the authors observed in the study, and (c) was symmetric, a condition different to the natural asymmetry of biological membranes. Thus, this work emphasizes that it is not just the presence of an l_o_ domain, but also its physicochemical characteristics and specific lipid components which condition nAChRs agglomeration.

With respect to neuronal nAChR, it was observed that α7 nAChR is associated with Chol-rich microdomains at somatic spine-rich regions of ciliary neurons and that the maintenance of these receptors within these domains is Chol-dependent (Brusés et al., 2001). Furthermore, in PC-12 cells, a rat pheochromocytoma cell line, α7 nAChR location in raft domains is necessary to regulate cAMP signal through the nicotinic activation, signaling that was altered by Chol depletion (Oshikawa et al., 2003). A similar relation between α7 nAChR location at raft domains and efficiently signaling, with a direct Chol influence, was also observed in CG neurons (Liu et al., 2008). Disruption of raft domains in the same CG neurons increased the mobility of α7 nAChRs in the synaptic space (Fernandes et al., 2010). Disruption of raft domains by removal of Chol and/or SM in rat primary hippocampal neurons slowed the kinetics of α7 nAChR desensitization through increasing the rate of recovery from desensitization and increased the agonist affinity and single-channel conductance (Colón-Sáez and Yakel, 2011). The authors observed the effects of raft domains disruption also on α3β2 nAChRs functionality. These results confirm that, as with muscle nAChR, neuronal nAChR functionality is modulated by its lipid microenvironment with the raft domains integrity a critical factor. On the contrary, α7 nAChR at non-neural tissues, in rat arterial endothelial (RAEC) and human venous endothelial (HUVEC), was found to occur in non-raft subcellular membrane fractions (Peña et al., 2011).

## Conclusion

**FIGURE 3:**
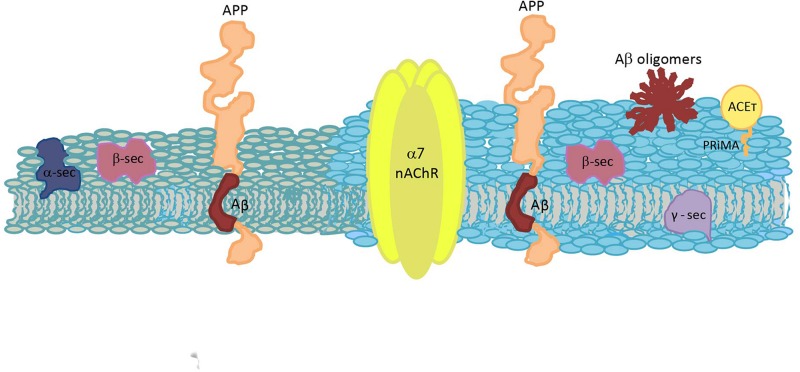
Schematic diagram of a plasma membrane, depicting the spatial relationship between the molecules involved in Aβ synthesis and the cholinergic system. Two different colors are used to represent a raft domain and a liquid-disordered domain (light blue and gray, respectively). APP, amyloid precursor protein; Aβ, amyloid β peptide; α7 nAChR, α7 nicotinic acetylcholine receptor; ACE_T_, tetrameric acetylcholinesterase; α-sec, α-secretase; β-sec, β-secretase; γ-sec, γ-secretase; and PRiMA, proline-rich membrane anchor.

Alzheimer’s disease is a progressive neurodegenerative condition, the etiopathogenic mechanisms of which are not totally understood. Due to its multifactorial character, the development of new drugs and effective treatments is still a challenge (Dineley, 2007). Here, we intended to focus only in the processes related to this disease that occur in the cell membrane, which allows to observe the multiple crosslinking between specific lipids and the membrane proteins involved in the amyloid process. In a dry human brain, half of its weight corresponds to lipids, molecules with great chemical diversity and complex dynamical heterogeneities (Piomelli et al., 2007). Thus, it is not surprising that through the years more and more biological functions are being related to them. Raft domains are implicated in several of the events involved in AD. Chol is a very important lipid at synaptic membranes (Barrantes, 2007) and it is also a principal author in AD, together with other lipids such as GM1, SM or PA. It is not surprising that APP, Aβ, nAChR and G4-PRiMA all have Chol-recognition amino acid sequences. Although there are still some controversies, there is no doubt that APP processing, Aβ production and Aβ action are intimately related to raft domains, and that the cholinergic system function is highly conditioned by both raft domains and Aβ. A continuous crosstalk between amyloid processing and cholinergic signaling occurs at physiological and pathological conditions, and shifting from one condition to the other is triggered by an imbalance in Aβ synthesis, being Chol homeostasis intimately implicated (Figure 3). Currently, the only available treatment for AD is a group of drugs that inhibit ACE. A better understanding of Aβ-α7 nAChR interactions and of the implication of Chol in particular, and membrane heterogeneities in general, could allow for a deepening of the understanding of this neurodegenerative pathology and could help define new therapeutic strategies and potential novel molecular targets.

The World Health Organization (WHO) declared dementia as a public health priority (in *Priority Medicines for Europe and the World “A Public Health Approach to Innovation”* by Saloni Tanna). The number of people worldwide with this condition is in continuous growth: whereas in 2010 this number was estimated to be 35.6 million, it is expected to be near 115.4 million in 2050, in line with the view that this number nearly doubles every 20 years. AD is the most common form of dementia and, hence, it has become a major public health problem because of the continuous increase in the age of the population (in fact, in 2050 it is expected that 22% of the world population will be aged 60 and over). Thus, it is imperative to count with specific biomarkers for early stages of the disease, to improve detection and evaluation and, of course, with effective therapies. At present, the only treatment available is symptomatic: ACE inhibitors, like physostigmine, tacrine, donepezil, rivastigmine, and galantamine. Although new knowledge is continuously emerging, until now and as suggested in this work, there is no consensus among the different coexisting hypotheses around this subject, several of which are antagonistic. This fact clearly contributes to the current situation: there is not a single specific AD treatment commercially available. A great variety of molecular targets were proposed for AD treatment, a few of which were explained here (like β and γ-secretases, α7 nAChR and ACE), and plenty of studies have been conducted on them. Much effort has been invested in this area, but more is still required. Science is facing a huge challenge. Further studies that contribute to the description and explanation of the AD etiopathology will be crucial for a final consensus on AD. Multitarget-drug design is an interesting strategy as AD involves a large number of different molecules. And finally, it should not be forgotten that membrane lipids are not just a “sea” where proteins function but, as explained in detail above, they are necessary for the proper function of these proteins. Chol, GM1, SM, among others, are important lipids for AChR function, conformation and membrane stabilization, and also for Aβ processing and Aβ-membrane insertion. Thus, lipid membrane perturbation, and hence, raft domains alteration and membrane signal perturbation, directly impact in several hot points of AD etiopathology and, for this reason, they can also be considered as interesting molecular targets for AD.

## Author Contributions

CF and SA contributed to the design, analysis, interpretation, and writing of the manuscript.

## Conflict of Interest Statement

The authors declare that the research was conducted in the absence of any commercial or financial relationships that could be construed as a potential conflict of interest.

## Abbreviations

Aβ: amyloid β protein
ACE: acetylcholinesterase
AD: Alzheimer’s disease
APP: amyloid precursor protein
BACE 1: β-site APP-cleaving transmembrane aspartic protease 1
CARC: inversed CRAC
Chol: cholesterol
CRAC: Chol recognition amino acid consensus
DPPC: dipalmitoylphosphatidylcholine
l_d_: liquid-disorder domain
LDM: low density membrane domain
l_o_: liquid-ordered domain
M1 to M4: nAChR transmembrane segments
MuSK: muscle specific receptor tyrosine kinase
nAChR: nicotinic acetylcholine receptor
NMJ: neuromuscular junction
PA: phosphatidic acid
PC: phosphatidylcholine
PE: phosphatidylethanolamine
PRiMA: proline-rich membrane anchor
PS1: presenilin 1
PUFA: polyunsaturated fatty acid
SM: sphingomyelin.
